# Alumni University of the Medical Department of the City of New York

**Published:** 1871-03

**Authors:** 


### Alumni University of the Medical Department of the City of New York.

The Executive Committee of the Alumni Association of the Medical Department of the University of the City of New York purpose the publication, at the earliest possible date, of a complete catalogue of the graduates from that institution since its foundation. The records of the Faculty having been destroyed in the burning of the college building some years ago, this project is one that should be seconded by every one of the alumni, of whom between two and three thousand are scattered throughout the United States. It is earnestly requested that each of these will without delay forward for enrolment his full name and post office address, with his professional history, including date of graduation, posts of honor and trust held, etc., and also any information which he may possess concerning former class mates who have since died or retired from practice. Communications should be addressed to the Secretary, Chas. Inslee Pardee, M. D., 72 West 35th street, New York.

